# Discovery of Curcuminoids as Pancreatic Lipase Inhibitors from Medicine-and-Food Homology Plants

**DOI:** 10.3390/nu16152566

**Published:** 2024-08-05

**Authors:** Xiao-Qin He, Hai-Dan Zou, Yi Liu, Xue-Jiao Chen, Atanas G. Atanasov, Xiao-Li Wang, Yu Xia, Siew Bee Ng, Maima Matin, Ding-Tao Wu, Hong-Yan Liu, Ren-You Gan

**Affiliations:** 1 Institute of Urban Agriculture, Chinese Academy of Agricultural Sciences, Chengdu National Agricultural Science & Technology Center, Chengdu 610213, China; 18883394393@163.com (X.-Q.H.); zellazou@foxmail.com (H.-D.Z.); liuyi03@caas.cn (Y.L.); xiayu01@caas.cn (Y.X.); 2 West China School of Public Health, West China Fourth Hospital, Sichuan University, Chengdu 610041, China; 3 College of Food and Bioengineering, Xihua University, Chengdu 610039, China; chenxuejiao@caas.cn (X.-J.C.); 15228782533@139.com (X.-L.W.); 4 Ludwig Boltzmann Institute Digital Health and Patient Safety, Medical University of Vienna, Spitalgasse 23, 1090 Vienna, Austria; atanas.atanasov@dhps.lbg.ac.at; 5 Institute of Genetics and Animal Biotechnology, The Polish Academy of Sciences, Jastrzebiec, 05-552 Magdalenka, Poland; m.matin@igbzpan.pl; 6 Singapore Institute of Food and Biotechnology Innovation (SIFBI), Agency for Science, Technology and Research (A*STAR), 31 Biopolis Way, Singapore 138669, Singapore; ngsb@sifbi.a-star.edu.sg; 7 Key Laboratory of Coarse Cereal Processing (Ministry of Agriculture and Rural Affairs), Sichuan Engineering & Technology Research Center of Coarse Cereal Industralization, School of Food and Biological Engineering, Chengdu University, Chengdu 610106, China; wudingtao@cdu.edu.cn; 8 Department of Food Science and Nutrition, Faculty of Science, The Hong Kong Polytechnic University, Kowloon, Hong Kong SAR, China

**Keywords:** medicine-and-food homology plants, pancreatic lipase, enzyme inhibition, affinity ultrafiltration, docking simulations

## Abstract

Researchers are increasingly interested in discovering new pancreatic lipase inhibitors as anti-obesity ingredients. Medicine-and-food homology plants contain a diverse set of natural bioactive compounds with promising development potential. This study screened and identified potent pancreatic lipase inhibitors from 20 commonly consumed medicine-and-food homology plants using affinity ultrafiltration combined with spectroscopy and docking simulations. The results showed that turmeric exhibited the highest pancreatic lipase-inhibitory activity, and curcumin, demethoxycurcumin, and bisdemethoxycurcumin were discovered to be potent pancreatic lipase inhibitors within the turmeric extract, with IC_50_ values of 0.52 ± 0.04, 1.12 ± 0.05, and 3.30 ± 0.08 mg/mL, respectively. In addition, the enzymatic kinetics analyses demonstrated that the inhibition type of the three curcuminoids was the reversible competitive model, and curcumin exhibited a higher binding affinity and greater impact on the secondary structure of pancreatic lipase than found with demethoxycurcumin or bisdemethoxycurcumin, as observed through fluorescence spectroscopy and circular dichroism. Furthermore, docking simulations supported the above experimental findings, and revealed that the three curcuminoids might interact with amino acid residues in the binding pocket of pancreatic lipase through non-covalent actions, such as hydrogen bonding and π-π stacking, thereby inhibiting the pancreatic lipase. Collectively, these findings suggest that the bioactive compounds of turmeric, in particular curcumin, can be promising dietary pancreatic lipase inhibitors for the prevention and management of obesity.

## 1. Introduction

Obesity is a chronic metabolic disease, one marked by an extreme accumulation of fat caused by excess calorie intake or disrupted fat metabolism, and it has become a major challenge for human health [1,2]. In addition, it has been reported that obesity may induce increasing risks of different chronic illnesses, such as type 2 diabetes, hyperlipidemia, osteoporosis, cardiovascular diseases, and even certain cancers [3,4,5,6]. Increasing evidence has revealed that blocking the digestion of dietary fat by inhibiting the digestive lipase is a potential strategy which can be used to alleviate obesity [7]. As a pivotal lipolytic enzyme, pancreatic lipase is responsible for the hydrolysis of most dietary fat into absorbable monotriglycerides and fatty acids, suggesting that inhibition of pancreatic lipase activity can reduce the systemic lipids, and the pancreatic lipase is a promising target for the prevention and management of obesity [1]. At present, orlistat is the only drug clinically approved as a pancreatic lipase inhibitor, but it has some side effects, such as abdominal pain, fecal urgency, and oily spotting [8]. Therefore, it is necessary to find novel, effective, and safe pancreatic lipase inhibitors among natural products for use in the alleviation of obesity.

Medicine-and-food homology plants have been traditionally and popularly used in people’s diets in some Asian countries, like China, for thousands of years, and have attracted increasing attention in modern times due to their various ingredients and multiple functions characterized by low toxicities and low levels of side effects [9,10]. For example, the mulberry leaf has been used to treat metabolic diseases like diabetes, dyslipidemia, obesity, atherosclerosis, and hypertension [11], and the extract of hawthorn berries has been reported to prevent hypertension and heart failure [12]. Thus, medicine-and-food homology plants can be potential natural sources for the discovery of potent natural pancreatic lipase inhibitors. In previous studies, certain medicine-and-food homology plants, like the mulberry leaf, had been reported to inhibit pancreatic lipase, but there was a lack of systematic investigation, and the inhibitory effects were not compared [13]. In addition, due to the complexity of compounds from medicine-and-food homology plants, the traditional screening method, that is, isolation and purification of each compound from plant extracts and then verification of their bioactivities, is cumbersome, labor-intensive, and time-consuming [9,14]. Alternatively, affinity screening by ultrafiltration coupled with high-performance liquid chromatography is an efficient technology used to rapidly filter and identify potential enzyme inhibitors from mixtures by evaluating the capacity of candidate molecules to bind with target proteins in a high-throughput manner [14,15,16]. With the advantages of higher efficiency, smaller sample size, and lower cost, it has become a popular technology for screening enzyme inhibitors in vitro [17]. In addition, various spectroscopic techniques and in silico docking simulations are frequently used to reveal the mechanisms of enzyme inhibitors based on the changes in characteristic spectra, functional group categories, and internal bond conformation of the compounds [9].

According to the Traditional Chinese Medicine theory, medicine and food share the same origin and may have similar functions in the prevention and treatment of various disease conditions; this is how the concept of medicine-and-food homology has been developed and popularized [18]. In the present work, affinity screening by ultrafiltration coupled with high-performance liquid chromatography was initially used to discover strong pancreatic lipase inhibitors from 20 commonly consumed medicine-and-food homology plants, which were selected based on the platform for querying special food information of the Special Food Safety Supervision and Administration Department of the State Administration for Market Regulation and Administration, China (Table S1). Then, the potential enzyme-inhibitory mechanisms of three main turmeric compounds with pancreatic lipase-inhibitory effects were elucidated by enzyme inhibition kinetics, fluorescence spectrum, circular dichroism, and docking simulation analyses.

## 2. Materials and Methods

### 2.1. Reagents and Preparation of Plant Extracts

The dried original samples of 20 medicine-and-food homology plants (listed in Table S1) were purchased from Guoxin Pharmacy (Beijing, China). The chemicals of pancreatic lipase from porcine pancreas type II, orlistat, and p-nitrophenyl butyrate were purchased from Sigma-Aldrich (St. Louis, MO, USA). The chemical standards of curcumin, demethoxycurcumin, and bisdemethoxycurcumin, were acquired from Chengdu RefMedic Biotech Co., Ltd. (Chengdu, China). A centrifugal ultrafiltration filter (AIMCO Ultra-0.5) was obtained from Merck Millipore (Burlington, NJ, USA). HPLC-grade formic acid and acetonitrile were purchased from TEDIA (Fairfield, CT, USA). All of the remaining reagents were analytically pure.

The method of preparing the raw plants was a slightly modified version of a method described in a previous study [9]. In brief, the raw materials of 20 medicine-and-food homology plants were crushed through a 60-mesh sieve (1.0 g) and extracted by an ultrasonic process at 40 °C for 4 h, with 10 mL 70% ethanol solution. After centrifugation of the composition (4000× *g*, 10 min), the liquid supernatant was collected and diluted to 15 mL with the 70% ethanol solution.

### 2.2. Pancreatic Lipase Inhibition with In Vitro Testing

The testing of the inhibitory effects of lipase activity was conducted as described in the literature, with some adjustments [19]. In sum, 100 μL plant extracts of different concentrations were added to 200 μL Tris-HCl buffer (50 mM, pH 7.4) and 100 μL pancreatic lipase solution (5 mg/mL). The composition was shaken well in a shaker and kept at 37 °C for 20 min. Afterward, 100 μL *p*-nitrophenyl butyrate (2 mM) was added to the composition and incubated for another 15 min at 37 °C. Lastly, the absorbance of the solution at 410 nm was tested immediately. The sample blank (A_sample blank_) and the control blank (A_control_) tests were conducted in the same way. The positive control was Orlistat. The inhibitory effect (%) was calculated with the below formula:

$$\mathrm{inhibition}\;(\%)=\frac{A_{\mathrm{control}}-(A_{\mathrm{sample}}-A_{\mathrm{sample blank}})}{A_{\mathrm{control}}}\times 100$$

### 2.3. Identification of Compounds in the Turmeric Extracts by UPLC-Q-TOF-MS

UPLC-Q-TOF-MS analysis was carried out by utilizing an Agilent 6545 LC/Q-TOF (Santa Clara, CA, USA). Briefly, an EclipsePlusC18 RRHD column (2.1 × 50 mm, 1.8 µm) was utilized. The mobile phases were 0.5% formic acid–water solution (solvent A) and methanol (solvent B). The high-resolution Q-Exactive Focus mass spectrometer was operated in positive mode, and the orbital trap was set to scan a range of *m*/*z* 100–1100. Data acquisition was performed using the Qualitative Analysis function of the Agilent MassHunter 10.0 software (Santa Clara, CA, USA). To analyze compounds in the turmeric extracts, the parent ions were compared with the database and the previous literature. The range of deviation between the calculated and observed *m*/*z* values was less than 5 ppm during the identification of individual compounds.

### 2.4. Affinity Screening by Ultrafiltration Coupled with HPLC

Affinity ultrafiltration screening was conducted based on methods described in the literature, along with slight modifications [15,16]. In sum, 100 μL pancreatic lipase (5 mg/mL), 100 μL turmeric extract, and 200 μL Tris-HCl buffer (50 mM, pH 7.4) were mingled and then kept at 37 °C for 20 min. After incubation, to intercept the pancreatic lipase–ligand complexes, the suspension was screened through a centrifugal tube with 10 kDa molecular weight cutoff, by centrifugation at 12,000× *g* for 20 min. Afterward, for the unbound constituents, the composition was rinsed three times with the above buffer and centrifugated at 12,000× *g* for 20 min. Then, 60% acetonitrile/water was added after two instances of centrifugation at 12,000× *g* for 20 min to separate ligands from their complexes, and a filtrate was obtained for HPLC analysis. The reaction containing pancreatic lipase inactivated at 100 °C for 10 min, instead of the original enzyme, was used as a negative control.

In the case of liquid chromatography analysis, 10 μL solution was injected into an HPLC and isolated with an Ultimate LP-C18 column (4.6 × 250 mm, 5 μm). The gradient elution program was as follows. The mobile phase was made up of 55% water containing 0.1% acetic acid (solvent A), and 45% acetonitrile (solvent B). Additionally, the column temperature, flow rate, and detection wavelength were set at 30 °C, 1 mL/min, and 425 nm, respectively.

### 2.5. Enzyme Inhibition Kinetics

Lineweaver–Burk analysis was used to identify the kinetic parameters and inhibition modes for the enzymatic reactions of curcumin, demethoxycurcumin, and bisdemethoxycurcumin against pancreatic lipase [20]. Briefly, the effects of *p*-nitrophenyl butyrate at different concentrations on the initial velocity (*v*) of different inhibitor concentrations was determined at a fixed concentration of pancreatic lipase (5 mg/mL) with reference to the above enzyme-activity assay method. The maximum catalytic reaction rate (*V_max_*), Michaelis constant (*K_m_*), and inhibition constant (*K_i_*) were determined within the double-reciprocal plot with the following equations:

$$\frac{1}{V}=\frac{K_{m}}{V_{max}}(1+\frac{\left[I\right]}{K_{i}})\times \frac{1}{\left[S\right]}+\frac{1}{V_{max}}$$

$$\mathrm{Slope}=\frac{K_{m}}{V_{\max}}+\frac{[I]\times K_{m}}{K_{i}\times V_{\max}}$$

The [*S*] represents concentrations of substrate, and [*I*] means inhibitor.

### 2.6. Fluorescence Spectrometry

The effects of curcumin, demethoxycurcumin, and bisdemethoxycurcumin on the tryptophan fluorescence spectra of pancreatic lipase were tested on an F-7000 fluorescence spectrometer based on methods described in earlier studies, with some modifications [15]. In brief, 3 mL of pancreatic lipase (1.0 × 10^−6^ M) was mixed with various concentrations of curcumin, demethoxycurcumin, and bisdemethoxycurcumin at 298 K and 310 K for 15 min. Then, using 280 nm as the excitation wavelength, the fluorescence emission spectrum of the mixed solution was measured in the range of 300 to 420 nm. The excitation and emission bandwidths were both stipulated to be 5 nm. The emission intensity was corrected for the inner optical filter effect [21]. The quenching rate constant (*K_q_*), Stern–Volmer dynamic quenching constant (*K_sv_*), binding constant (*K_a_*), binding site (*n*), and thermodynamic parameters (Δ*H* and Δ*S*) were calculated with the Stern–Volmer equation:

$$\frac{F_{0}}{F}=1+K_{\mathrm{sv}}\left[I\right]=1+K_{q}\tau_{0}[I]$$

$$\mathrm{lg}[(F_{0}-F)/F=\mathrm{lg}K_{a}+n\mathrm{lg}\left[I\right]$$

$$\ln K_{a}=-\frac{1}{T}[\frac{\Delta H}{R}]+\frac{\Delta S}{R}$$

where *F*_0_ and *F* are the fluorescence intensities in the presence and absence of inhibitor, respectively. [*I*], *τ*_0,_ Δ*H*, Δ*S*, and *R* represent the concentration of inhibitors, the constant of the lifetime of the fluorophore (10^−8^ s), the variation in enthalpy of the system, the variation in entropy of the system, and the gas constant of 8.31 J (mol/K), respectively.

### 2.7. Circular Dichroism Measurement

A Chirascan plus circular dichroism spectrophotometer (Applied Photophysics, London, UK) was used for pancreatic lipase secondary structure measurement in cells with a 1.0 mm path length, as reported in the literature [4]. Simply, pancreatic lipase was diluted to 1.0 × 10^−6^ M in PBS (0.1 M, pH 7.4), and the circular dichroism spectra were tested from 200 to 250 nm when pancreatic lipase was mixed with curcumin, demethoxycurcumin and bisdemethoxycurcumin at 37 °C for 5 min at the molar ratios of 1:1, respectively. The response time was set to 1 nm while the bandwidth was set to 2 nm. The background signal of the pancreatic lipase-free solution was subtracted, and every circular dichroism spectrum was the cumulation of triplicate scans. Finally, the secondary structure of the protein was identified with the SELCON3 method on DichroWeb [22].

### 2.8. Molecular Docking Simulation

The MOE software version 20180101 was used to determine the specific interactions of curcumin, demethoxycurcumin, bisdemethoxycurcumin, and *p*-nitrophenyl butyrate with pancreatic lipase. The 2D structures of three small molecules and *p*-nitrophenyl butyrate were downloaded in the SDF format from PubChem, and the structure of pancreatic lipase (PDB ID: 1ETH) was downloaded in the PDB format from Protein Data Bank. The water, salts and ligands of 1ETH were removed, and then all hydrogen atoms and charges were added. The lowest score of the binding conformation was selected for analysis [16].

### 2.9. Statistical Analysis

The data was expressed as mean ± standard deviation. Using ANOVA and the post hoc Duncan test, the data was analyzed using SPSS 20.0 software (SPSS Inc., Chicago, IL, USA), with statistical significance defined at *p* < 0.05.

## 3. Results and Discussion

### 3.1. Pancreatic Lipase-Inhibitory Activities of the Extracts of 20 Medicine-and-Food Homology Plants

Pancreatic lipase is an important lipolytic enzyme in lipid absorption and plays an indispensable role in the whole digestion process. In addition, the functional components of many homologous Chinese medicines also show potential anti-obesity effects. In this study, the pancreatic lipase-inhibitory activities of 20 commonly consumed medicine-and-food homology plants were determined. The results are exhibited in Table 1; the IC_50_ values of 20 plants range from 9.70 to 294.30 mg/mL, among which turmeric showed the strongest inhibitory effect (9.70 ± 0.29 mg/mL), followed by hawthorn fruit (23.18 ± 0.81 mg/mL), lotus leaf (38.74 ± 0.34 mg/mL), sea buckthorn fruit (43.73 ± 1.47 mg/mL), and mulberry (45.96 ± 3.02 mg/mL). Podsedek et al. [19] determined that blueberry extract was a potent pancreatic lipase inhibitor (IC_50_ = 14.53 g/L), and was more effective than the extracts from kiwi, pomegranate, raspberry, or bilberry (IC_50_ = 16.12, 18.53, 22.54, and 25.12 mg/mL, respectively). In addition, in the course of pancreatic lipase research on various natural products, the methanol extracts of muscadine skin had one of the most significant pancreatic lipase-inhibitory effects, with the IC_50_ value at 11.15 mg/mL [23]. Compared with the previous study, the turmeric extract exhibited a better inhibitory effect and might be a more promising inhibitor of pancreatic lipase. Therefore, turmeric extract was selected for further ultrafiltration screening.

### 3.2. Identification and Quantification of Major Compounds in the Turmeric Extracts

UPLC-Q-TOF-MS analysis was carried out to characterize the major compounds in the turmeric extracts. As shown in Table 2, a total of 31 compounds were tentatively identified from the turmeric. Their retention times, formulas, calculated *m*/*z*, and observed *m*/*z* are summarized in Table 2. Based on the previous literature and database searches, these compounds were tentatively identified as L-valine, L-pyroglutamic acid, adenosine, 5-hydroxymethyl-2-furaldehyde, vanillin, 4′-hydroxyacetophenone, isoscopoletin, genipin, 4′-demethylepipodophyllotoxin, neohesperidin, arctigenin, apigenin 7-O-β-D-glucuronide, dimethoxycurcumin, carvacrol, tanshinone II A, (-)-8′-epi-aristoligone, xanthohumol, 1,2-dimethoxy-4-(1-propenyi)benzene, neocnidilide, curdione, bisdemethoxycurcumin, Rel-(8R,8′R)-dimethyl-(7S,7′R)-bis(3,4-methylenedioxyphenyl)tetrahydro-furan, (-)-deoxypodophyllotoxin, demethoxycurcumin, glycocholic acid, curcumin, glycycoumarin, batatasin I, orientin 2″-O-p-trans-coumarate, poricoic acid B, and liquidambaric acid. Among them, the three compounds with the highest relative content were bisdemethoxycurcumin, demethoxycurcumins, and curcumin, so these three compounds were selected for further HPLC experiments.

### 3.3. Online Screening of Pancreatic Lipase Inhibitors from the Turmeric Extract

**Figure 1:**
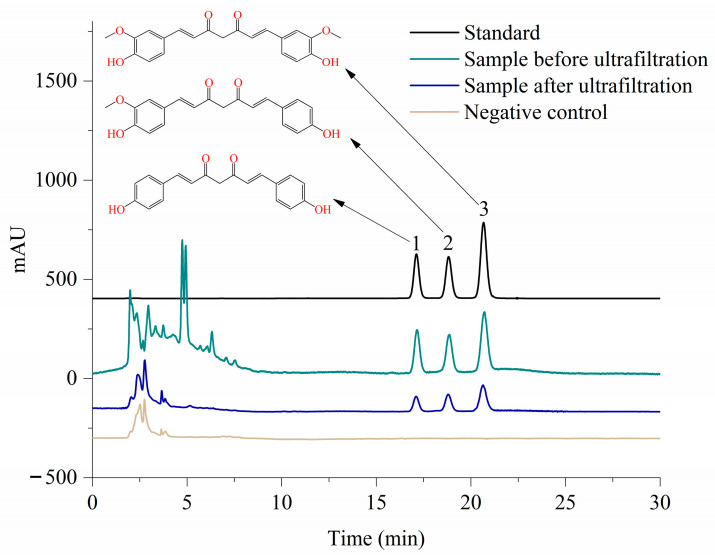
Affinity ultrafiltration, coupled with HPLC, of potential pancreatic lipase ligands from turmeric extract at 425 nm. Peak 1, bisdemethoxycurcumin; Peak 2, demethoxycurcumin; Peak 3, curcumin.

Not all plant compounds can be considered effective inhibitors of pancreatic lipase. Therefore, screening and identifying bioactive ingredients from extract mixtures are important measures for the precise utilization of plant extracts [24]. Affinity ultrafiltration is a general method for high-throughput screening of potential enzyme inhibitors from natural plants which mainly consists of four steps: incubating the sample with the enzyme to bind the ligand to the target protein, separating the protein–ligand complex from the unbound small molecules by ultrafiltration, releasing the ligand by destroying the protein–ligand complex with the addition of an organic solvent, and detecting specific binding chemicals [14,15]. Therefore, the potential compounds in turmeric extracts that might specifically bind to pancreatic lipase were further screened and identified by affinity ultrafiltration coupled with HPLC. As the main chemical components of turmeric, curcumin, desmethoxycurcumin, and hyperploxycurcumin play important roles in the of various bioactivities of turmeric. As illustrated in Figure 1, compounds **1**, **2**, and **3** were found to specifically bind with pancreatic lipase, contrasted with the negative control of the chromatogram, which was identified as bisdemethoxycurcumin, demethoxycurcumin and curcumin, respectively, given the contrast in retention time in the results from the HPLC. Moreover, the changes of peak area could preliminarily reflect the binding rate of inhibitors to pancreatic lipase [16], and the binding rates of bisdemethoxycurcumin, demethoxycurcumin, and curcumins to pancreatic lipase were 41.37%, 49.33%, and 54.69%, respectively. These results indicated that bisdemethoxycurcumin, demethoxycurcumin, and curcumin might be potential pancreatic lipase inhibitors within the turmeric extract, and their binding capacity was bisdemethoxycurcumin < demethoxycurcumin < curcumin.

### 3.4. Inhibitory Effects and Mechanisms of the Promising Inhibitors on Pancreatic Lipase

**Figure 2:**
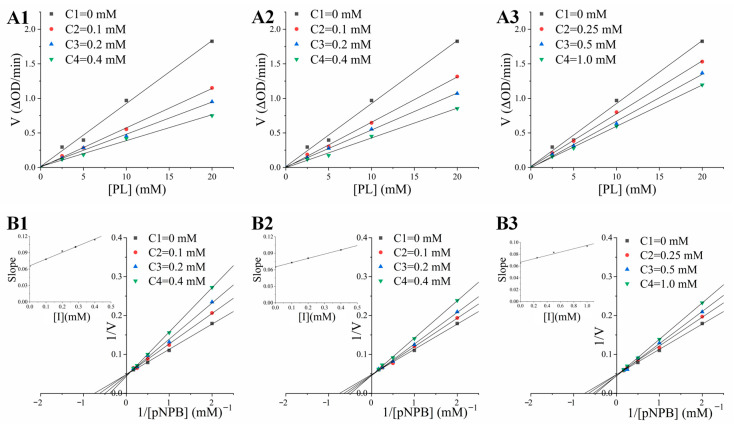
Inhibitory effects and mechanisms of the promising inhibitors of pancreatic lipase. (**A**) Reversibility of curcumin (**A1**), demethoxycurcumin (**A2**), and bisdemethoxycurcumin (**A3**) on pancreatic lipase inhibition; (**B**) The Lineweaver–Burk double curves of curcumin (**B1**), demethoxycurcumin (**B2**), and bisdemethoxycurcumin (**B3**) against pancreatic lipase.

The inhibitory effects of curcumin, demethoxycurcumin, and bisdemethoxycurcumin on pancreatic lipase were further verified. The IC_50_ values of curcumin, demethoxycurcumin, and bisdemethoxycurcumin were 0.52 ± 0.04 mg/mL, 1.12 ± 0.05 mg/mL, and 3.30 ± 0.08 mg/mL, as shown in Table 3, indicating that curcumin possessed a stronger inhibition on pancreatic lipase than those of demethoxycurcumin or bisdemethoxycurcumin, which was fitted with the determinations of affinity ultrafiltration coupled with HPLC. Previous studies showed that multiple polyphenols are potent inhibitors of pancreatic lipase [4]. Pu et al. (2023) [25] found that banana condensed tannins could significantly enhance the inhibition of pancreatic lipase with a lower IC_50_ value at 0.55 mg/mL. In addition, the findings by Proençai et al. [26] revealed that chiisanoside showed a strong inhibitory effect on pancreatic lipase activity, with the IC_50_ value at 0.74 mg/mL. The inhibitory patterns of curcumin, demethoxycurcumin, and bisdemethoxycurcumin on pancreatic lipase were further studied by means of enzyme inhibition kinetics. The correlation between the initial velocity (*v*) and the concentration of pancreatic lipase in the presence of different concentrations of curcumin (Figure 2A1), demethoxycurcumin (Figure 2A2), and bisdemethoxycurcumin (Figure 2A3) was a series of straight lines passing through the origin, and the slope declined with the increasing density of the inhibitors. These results manifested that the inhibitory effects of curcumin, demethoxycurcumin, and bisdemethoxycurcumin on pancreatic lipase were reversible, and that the substances might interact with pancreatic lipase through the formation of non-covalent intermolecular bonds [20]. Additionally, there was a set of straight lines that intersected in the first quadrant, and the slope of the lines increased with the raising density of curcumin (Figure 2B1), demethoxycurcumin (Figure 2B2), and bisdemethoxycurcumin (Figure 2B3), while the *V_max_* of reaction remained unchanged. These results indicated that curcumin, demethoxycurcumin, and bisdemethoxycurcumin belonged to a group of competitive inhibitors that have either one or a group of binding sites in the pancreatic lipase active center. That is, these three inhibitors might reduce the catalytic activity of pancreatic lipase by directly competing with the substrate for the enzyme’s active site, thus exhibiting negative effects, which is consistent with the determinations of Li [20]. Moreover, the dissociation constant *K_i_* of the inhibitor–enzyme complex plays a vital part in evaluating the affinity between the enzyme and the inhibitor, and a smaller *K_i_* value indicates a stronger binding effect of the enzyme and the inhibitor [4]. In this study, the *K_i_* values of curcumin, demethoxycurcumin, and bisdemethoxycurcumin were 0.45 ± 0.07, 0.84 ± 0.04, and 2.34 ± 0.11 mM, respectively, which determined that the curcumin possessed a stronger catalytic affinity for pancreatic lipase than did the demethoxycurcumin and bisdemethoxycurcumin.

### 3.5. Fluorescence Spectroscopy

#### 3.5.1. Fluorescence-Quenching Spectrum Analysis

**Figure 3:**
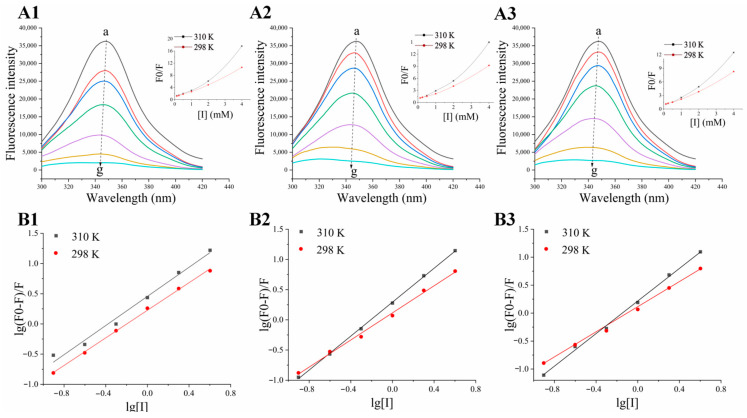
The fluorescence-quenching effects of the promising inhibitors on pancreatic lipase. (**A**) Fluorescence spectra of pancreatic lipase in the presence of curcumin (**A1**), demethoxycurcumin (**A2**), and bisdemethoxycurcumin (**A3**) on pancreatic lipase (the inset at upper right shows the corresponding Stern-Volmer plots between inhibitors and pancreatic lipase at 298 K and 310 K). Concentration of inhibitors in a–g was 0, 0.125, 0.25, 0.5, 1, 2, and 4 mM. (**B**) Double logarithm plot of curcumin (**B1**), demethoxycurcumin (**B2**), and bisdemethoxycurcumin (**B3**): quenching effects on pancreatic lipase fluorescence at 298 K and 310 K.

Fluorescence spectroscopy is a prospective technology used to research the interaction mode between small molecules and proteins as it reflects the difference in the microenvironments of protein fluorophores, and it has been commonly applied in the structural investigation of functional proteins [4]. There are three aromatic amino acids with intrinsic fluorescent groups present in pancreatic lipase, including residues of phenylalanine, tyrosine, and tryptophan, in which tryptophan produces the primary fluorescence [4,24]. Additionally, it has been reported that the fluorescence intensity can be reduced due to various intermolecular interactions, consisting of molecular rearrangements, ground state complex formation, etc. [27]. Since the variation in the intrinsic fluorescence intensity of pancreatic lipase can indicate the stage of amino acid residues and the ambient environment, it provides conformational information about the intermolecular binding between pancreatic lipase and its inhibitors [28]. The fluorescence emission spectra of pancreatic lipase in the presence of curcumin, demethoxycurcumin, and bisdemethoxycurcumin at the excitation wavelength of 280 nm are given in Figure 3. The results show a clear fluorescence emission peak at around 350 nm that belonged to the intrinsic fluorescence of tryptophan residues situated in the protein’s interior [29]. Additionally, with the increased concentration of curcumin (Figure 3A1), demethoxycurcumin (Figure 3A2), and bisdemethoxycurcumin (Figure 3A3), the fluorescence intensity of pancreatic lipase was notably reduced, demonstrating that the endogenous fluorescence of pancreatic lipase could be effectively quenched by curcumin, demethoxycurcumin, and bisdemethoxycurcumin, a phenomenon identical with the results of pancreatic lipase activity inhibition. Moreover, the addition of curcumin, demethoxycurcumin, and bisdemethoxycurcumin also prompted an obvious blueshift in the maximum emission peak of pancreatic lipase, from 350 nm to 342 nm, suggesting that curcumin, demethoxycurcumin, and bisdemethoxycurcumin might make the protein fold, transforming the microenvironment of tryptophan residues into a more hydrophobic environment [25,30]. The above results indicated that curcumin, demethoxycurcumin, and bisdemethoxycurcumin might interact with fluorophore at the pancreatic lipase active site or other sites, such as those associated with tryptophan residues.

#### 3.5.2. Fluorescence-Quenching Type and Binding Site Number Analysis

With the Stern–Volmer equation, the fluorescence-quenching mechanisms of curcumin, demethoxycurcumin, and bisdemethoxycurcumin on pancreatic lipase can be investigated. In general, the fluorescence-quenching type is categorized as a single or hybrid quenching, in which *F*_0_/*F* is the linear relationship to [*I*] in single quenching, while mixed quenching is a curve along the *y*-axis [4]. The Stern–Volmer plots of curcumin, demethoxycurcumin, and bisdemethoxycurcumin (Figure 3A3) showed obvious upward curves relative to the *y*-axis, suggesting the presence of a mixed quenching process at high concentrations. Additionally, the *K_q_* value is used to evaluate the effects of quenching agents on fluorescent groups [30]. According to Table 4, the *K_q_* values of curcumin, demethoxycurcumin, and bisdemethoxycurcumin increased with increasing temperature, but all of them were far beyond the maximum dynamic quenching constant (2.0 × 10^10^ L·mol^−1^·s^−1^), which further showed that the mechanisms of these three molecules belonged to a combined quenching attributed to two kind of formations, static and dynamic collision [24,27], with static quenching possibly occupying a dominant position [4]. Moreover, the *K_q_* values of curcumin were higher than those of demethoxycurcumin and bisdemethoxycurcumin, suggesting that curcumin had stronger pancreatic lipase-quenching ability than the other two compounds. 

Additionally, the *K_a_* and binding sites (n) of the three compounds to pancreatic lipase were estimated from the linear plot of lg[(*F*_0_ − *F*)/*F*] versus lg[*I*]. The sequence of *K_a_* values of the three inhibitors on pancreatic lipase was curcumin > demethoxycurcumin > bisdemethoxycurcumin > 1.0 × 10^4^ L·mol^−1^, as shown in Table 4, indicating that curcumin had the highest binding efficiency on pancreatic lipase. In addition, the values of the binding sites of curcumin, demethoxycurcumin, and bisdemethoxycurcumin were approximately equal to 1, exhibiting that there was only one binding site between those three inhibitors and pancreatic lipase [4], a finding which matched the results from enzyme kinetics. Generally, our results indicated that these three compounds could quench the inherent fluorescence of pancreatic lipase, and curcumin showed the strongest affinity, which might be attributed to the formation of a ground-state complex between the fluorescence group at the pancreatic lipase active site and curcumin, thereby reducing the capacity of pancreatic lipase to degrade substrates.

#### 3.5.3. Thermodynamic Parameter and Binding Force Analysis

Many tiny organic molecules and proteins are generally merged into the complex through non-covalent interactions [7,24]. Thermodynamic parameters can provide information about the types of interactions between molecules and the spontaneity of reactions [31]. Therefore, the Δ*H*, Δ*S*, and Δ*G* were calculated by using the Van’t Hoff equations to identify the detailed types of binding force in evidence between the inhibitors and pancreatic lipase. As illustrated in Table 3, the negative Δ*G* in all groups showed that the interaction of different inhibitors with pancreatic lipase was unsolicited. Moreover, both Δ*H* and Δ*S* < 0 showed that the van der Waals forces and hydrogen bonds might be effective in the interaction between those three inhibitors and pancreatic lipase [32], a determination which would be consistent with the results of a previously published report [24].

### 3.6. Circular Dichroism Analysis

**Figure 4:**
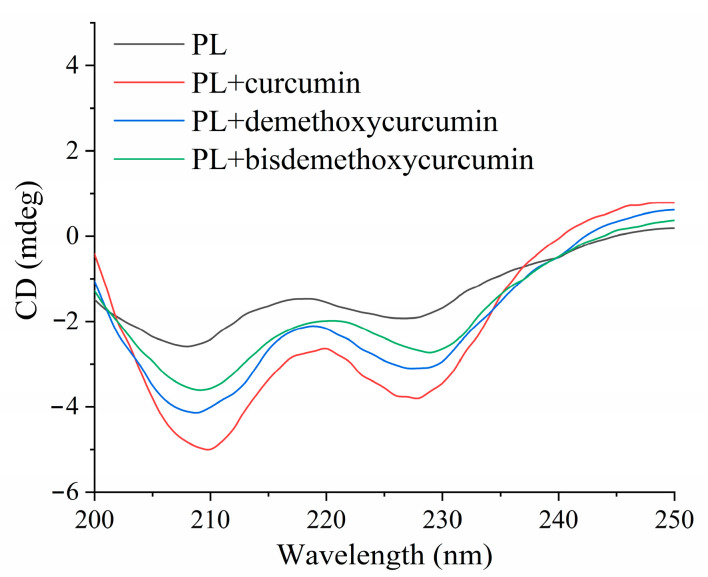
Circular dichroism spectrum of pancreatic lipase and curcumin, demethoxycurcumin, and bisdemethoxycurcumin.

Far-UV circular dichroism is widely used for assessing the conformation of protein secondary structure and side chain environment, which can reflect the structural changes of protein α-helix, β-fold, β-turn, and random coil [33]. The effects of curcumin, demethoxycurcumin, and bisdemethoxycurcumin on the secondary structure of pancreatic lipase are exhibited in Figure 4. Two apparent negative peaks are shown at the wavelengths of 208 and 228 nm, which could be due to the n → π* transition of the α-helix structure [31]. Additionally, the intensities of both absorption peaks are obviously raised after the addition of curcumin, demethoxycurcumin, and bisdemethoxycurcumin, indicating that the characteristics of pancreatic lipase’s secondary structure were changed. Moreover, the specific percents of α-helix, β-fold, β-turn, and random coil obtained from the spectrum are shown in Table 4. Under the effects of curcumin, demethoxycurcumin, and bisdemethoxycurcumin, the α-helix content of pancreatic lipase increased by 93.78%, 49.15%, and 20.34%, respectively, while the β-sheet was decreased by 39.76%, 33.46%, and 14.57%, respectively. Homologous outcomes have also been obtained in the previous literature; that is, the addition of persimmon tannin [21], (−)-epigallocatechin-3-gallate [34], cyanidin-3-O-glucoside, and catechin [16] significantly increased the proportion of α-helix structures and affected the activity of pancreatic lipase. As the active pocket region of pancreatic lipase mainly consists of α-helix structures, the enhancement in α-helix content can influence the active site of pancreatic lipase by reducing the open conformation, thus making it more difficult for the substrate to access the active center [27,33]. This might be part of the reason why curcumin had a greater inhibitory effect on pancreatic lipase than did demethoxycurcumin or bisdemethoxycurcumin. Therefore, these results further indicated that the interactions of these three inhibitors with pancreatic lipase caused conformational changes of pancreatic lipase to a tighter structure, which resulted in a reduction in enzyme-catalytic activity. 

### 3.7. Docking Simulation Analysis

**Figure 5:**
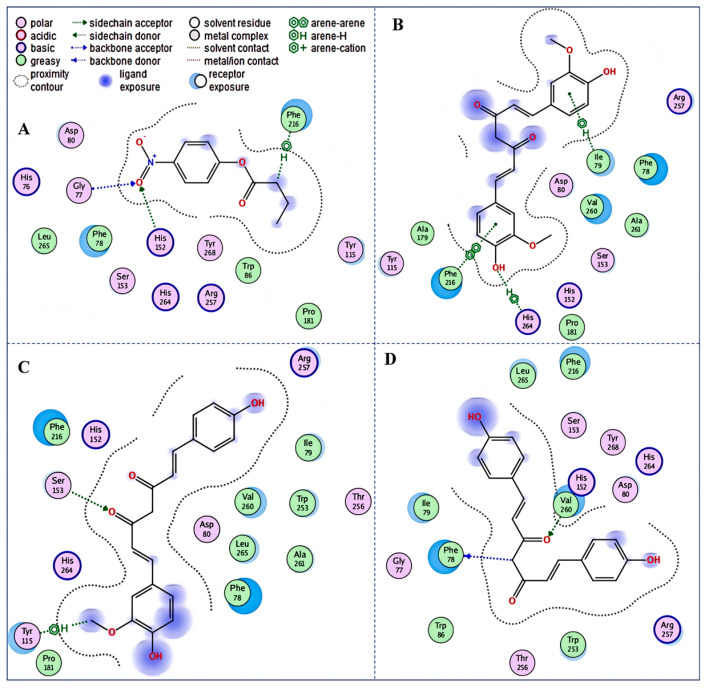
Docking simulation conformations of the promising inhibitors with pancreatic lipase. The 2-dimensional diagrams of (**A**) *p*-nitrophenyl butyrate, (**B**) curcumin, (**C**) demethoxycurcumin, and (**D**) bisdemethoxycurcumin interacting with pancreatic lipase.

**Figure 6:**
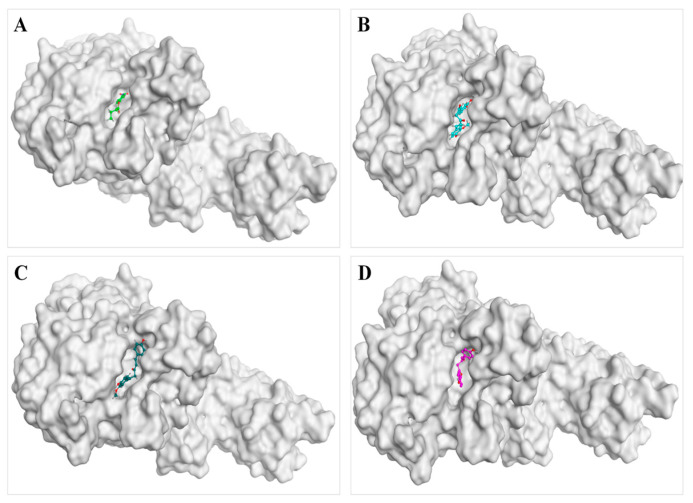
Preferred docking positions of (**A**) *p*-nitrophenyl butyrate, (**B**) curcumin, (**C**) demethoxycurcumin, (**D**) and bisdemethoxycurcumin on pancreatic lipase. *p*-nitrophenyl butyrate is colored in bright green; curcumin is colored in light blue; demethoxycurcumin is colored in dark green; bisdemethoxycurcumin is colored in purple; surrounding residues in the binding pocket are colored in red.

As a theoretical simulation approach, the docking simulation is commonly applied to explore the potential interactions between proteins and small molecules, which can provide some information about intermolecular binding patterns and affinity based on molecular structure [30]. Thus, an additional explanation for the inhibitory mechanisms of curcumin, demethoxycurcumin, and bisdemethoxycurcumin on pancreatic lipase from the perspective of the ligand–enzyme interaction was investigated by docking simulations. As the spatial conformation of the inhibitor–enzyme complex shown in Figure 5A, the substrate *p*-nitrophenyl butyrate was observed to bind to His152, Phe216, and Gly77 residues of pancreatic lipase with the docking scores of −1.1 kcal/mol, −0.6 kcal/mol, and −1.0 kcal/mol, respectively, which was consistent with the previously reported enzyme–substrate binding sites [20]. In addition, curcumin was observed to be enclosed by 13 amino acid residues of PL, of which Phe216 (−1.6 kcal/mol), His264 (−1.7 kcal/mol), and Ile79 (−0.6 kcal/mol) residues formed one π-π interaction and two π-H bond interactions with curcumin, respectively (Figure 5B). Similarly, both demethoxycurcumin and bisdemethoxycurcumin were encircled by 15 amino acid residues, in which hydrogen bond and π-H bond interactions were observed between demethoxycurcumin and Ser153 (−1.2 kcal/mol) and Tyr115 (−0.5 kcal/mol) residues, respectively (Figure 5C), while between bisdemethoxycurcumin and Phe 78 (−0.7 kcal/mol) and His 152 (−0.6 kcal/mol) (Figure 5D), there were two hydrogen bonds formed. It has been reported that the active catalytic site of pancreatic lipase was a trypsin-like catalytic triad composed of Ser 153, Asp 176, and His 263 [35]. In this study, it was obvious that curcumin, demethoxycurcumin, and bisdemethoxycurcumin all bond with the amino acid residues of the active catalytic sites and substrate binding sites of pancreatic lipase (Figure 6) through hydrogen bonding and, to a certain extent, hydrophobic interaction, revealing that these three inhibitors might weaken the catalytic activity of pancreatic lipase by entering the hydrophobic cavity to occupy the active site and stop the substrate from entering the active center to interact with pancreatic lipase [20]; this is related to the finding of the enzyme kinetics study, in which the inhibitory type was determined to be competitive inhibition. Furthermore, the interactions of curcumin, demethoxycurcumin, and bisdemethoxycurcumin with the endogenous fluorescent amino acids Phe 215, Ser 153, and His 152 of pancreatic lipase, respectively, also resulted in findings consistent with the results mentioned before.

Generally, lower docking simulation energy indicated the formation of a more stable ligand–protein complex [16]. In this manuscript, the docking energy of curcumin to pancreatic lipase was higher than those of demethoxycurcumin and bisdemethoxycurcumin (−7.05 kcal/mol, −6.68 kcal/mol and −6.44 kcal/mol, respectively), indicating that curcumin bonds pancreatic lipase more easily and closely, which was consistent with the IC_50_ values. The inhibitory or binding ability of natural compounds such as polyphenols and flavonoids on pancreatic lipase were highly connected with the structural properties of the former. Huang [4] contrasted the inhibitory effects of four polymethoxylated flavones on lipase and found that the increase of the inhibitory potential of lipase was related to the quantity and site of methoxy group, and a higher number of methoxy resulted in a stronger enzyme-inhibition activity. In addition, Kawaguchi et al. [36] reported that the hydroxy function in position 3′ (R2) and methoxy function in position 4′ (R3) appear to favor pancreatic lipase-inhibitory activity. Moreover, Buchholz and Melzig [37], in a review, found that the molecular size and the number of methoxy and hydroxyl groups all could notably affect the binding of polyphenols to pancreatic lipase. The results of this study also supported the above views; that is, the large number of methoxy groups in curcumin might contribute positively to the inhibition of pancreatic lipase inhibitors.

## 4. Conclusions

In this paper, the pancreatic lipase inhibition of 20 common medicine-and-food homology plants was evaluated through in vitro experiments, and it was found that turmeric showed the highest pancreatic lipase-inhibitory potential. Subsequently, three compounds, curcumin, demethoxycurcumin, and bisdemethoxycurcumin, with IC_50_ values of 0.52 ± 0.04 mg/mL, 1.12 ± 0.05 mg/mL, and 3.30 ± 0.08 mg/mL, respectively, were further identified as effective pancreatic lipase-inhibitory components of turmeric extract by an ultrafiltration LC screening strategy. Moreover, from the perspective of enzyme inhibition kinetics, spectroscopy, and docking simulations, it was further revealed that the inhibitory mechanism of curcumin, demethoxycurcumin, and bisdemethoxycurcumin on pancreatic lipase was reversible competitive inhibition, which could effectively quench the intrinsic fluorescence of pancreatic lipase and noticeably change its secondary structure through the formation of non-covalent interactions like hydrogen bonding and π effects. Finally, structure–activity relationship analysis indicated that the principal pancreatic lipase-inhibitory activity of curcumin might be connected with the number of methoxyl groups. In sum, this research provided basic data supporting the potential of turmeric and its active components, especially curcumin, demethoxycurcumin, and bisdemethoxycurcumin, as pancreatic lipase inhibitors, a finding which might contribute to their application in anti-obesity products, such as functional foods or pharmaceuticals.

## Figures and Tables

**Table 1:** Pancreatic lipase-inhibitory activities of 20 medicine-and-food homology plants.

Samples	IC_50_ (mg of Dried Raw Powder/mL)
Orlistat	0.36 ± 0.12 ^a^
Turmeric	9.70 ± 0.29 ^b^
Hawthorn Fruit	23.18 ± 0.81 ^c^
Lotus Leaf	38.74 ± 0.34 ^c^
Seabuckthorn Fruit	43.73 ± 1.47 ^c^
Mulberry Fruit	45.96 ± 3.02 ^c^
Palmleaf Raspberry Fruit	62.94 ± 2.70 ^d^
Lesser Galangal Rhizome	65.71 ± 0.32 ^d^
Solomonseal Rhizome	76.67 ± 1.76 ^e^
Radish Seed	92.91 ± 0.38 ^f^
Mulberry Leaf	107.95 ± 1.08 ^g^
Dandelion	132.22 ± 2.49 ^h^
Smoked Plum	142.83 ± 4.83 ^h^
Citron Fruit	150.41 ± 1.12 ^h^
Eucommia Bark	152.47 ± 6.75 ^i^
Hyacinth Beau	172.29 ± 2.67 ^j^
Bulbus Lilii	214.58 ± 4.06 ^k^
Germinated Barley	243.70 ± 3.99 ^l^
Indian Bread	282.38 ± 1.15 ^l^
Tall Gastrodia Tuber	284.84 ± 4.70 ^lm^
Cassia Seed	294.30 ± 6.26 ^m^

^a–m^ letters demonstrate statistical significance (*p* < 0.05).

**Table 2:** Tentative identification of compounds in the turmeric extract by UPLC-Q-TOF-MS.

No.	Formula	Retention Time (min)	Mass	Observed [M + H]^+^	Error (ppm)	Identified Compounds
1	C_5_H_11_NO_2_	0.48	117.0791	118.0864	1.35	L-Valine ^a^
2	C_5_H_7_NO_3_	0.766	129.0426	130.0498	0.11	L-Pyroglutamic acid ^a^
3	C_10_H_13_N_5_O_4_	1.175	267.0971	268.1044	1.47	Adenosine ^ab^
4	C_6_H_6_O_3_	2.451	126.032	127.0393	2.46	5-Hydroxymethyl-2-Furaldehydea
5	C_8_H_8_O_3_	5.718	152.0474	153.0547	0.63	Vanillin ^ab^
6	C_8_H_8_O_2_	6.266	136.0523	137.0596	−1.07	4′-Hydroxyacetophenone ^a^
7	C_10_H_8_O_4_	6.963	192.0426	193.0499	1.54	Isoscopoletin ^a^
8	C_11_H_14_O_5_	7.617	226.0846	227.0919	2.08	Genipin ^a^
9	C_21_H_20_O_8_	7.955	400.116	401.1232	0.38	4′-Demethylepipodophyllotoxin ^a^
10	C_28_H_34_O_15_	10.505	610.1891	611.1967	−1.15	Neohesperidin ^a^
11	C_21_H_24_O_6_	11.425	372.1577	373.1648	1.02	Arctigenin ^a^
12	C_21_H_18_O_11_	11.701	446.085	447.0923	0.3	Apigenin 7-O-beta-D-glucuronide ^a^
13	C_23_H_24_O_6_	12.551	396.1555	397.1629	0.55	Dimethoxycurcumin ^ab^
14	C_10_H_14_O	13.297	150.1044	151.1117	−0.51	Carvacrol ^ab^
15	C_19_H_18_O_3_	15.585	294.1258	295.1329	0.63	Tanshinone II A ^a^
16	C_22_H_26_O_5_	15.612	370.1778	371.1852	−0.61	(-)-8′-epi-Aristoligone ^a^
17	C_21_H_22_O_5_	15.807	354.147	355.1542	0.83	Xanthohumol ^ab^
18	C_11_H_14_O_2_	16.154	178.0995	179.1068	0.84	1,2-Dimethoxy-4-(1-propenyi) benzene ^a^
19	C_12_H_18_O_2_	16.941	194.13	195.1373	−3.42	Neocnidilide ^a^
20	C_15_H_24_O_2_	16.993	236.178	237.1852	1.37	Curdione ^ab^
21	C_20_H_20_O_5_	17.016	340.1315	341.1387	1.22	Rel-(8R,8′R)-dimethyl-(7S,7′R)-bis (3,4-methylenedioxyphenyl)tetrahydro-furan ^a^
22	C_19_H_16_O_4_	17.215	308.1049	309.1121	−0.01	Bisdemethoxycurcumin ^ab^
23	C_22_H_22_O_7_	17.258	398.1365	399.1438	−0.02	(-)-Deoxypodophyllotoxin ^a^
24	C_20_H_18_O_5_	17.261	338.1156	339.1229	−4.5	Demethoxycurcumin ^ab^
25	C_26_H_43_NO_6_	17.277	465.3089	466.3165	−0.23	Glycocholic acid ^a^
26	C_21_H_20_O_6_	17.308	368.126	369.1333	0.04	Curcumin ^ab^
27	C_21_H_20_O_6_	17.308	368.126	369.1333	0.04	Glycycoumarin ^a^
28	C_17_H_16_O_4_	17.308	284.1045	285.1118	−1.12	Batatasin I ^a^
29	C_30_H_26_O_13_	17.712	594.1374	595.1444	0.13	Orientin 2″-O-p-trans-coumarate ^a^
30	C_30_H_44_O_5_	18.208	484.3193	485.3266	0.82	Poricoic acid B ^a^
31	C_30_H_46_O_3_	18.866	454.3446	455.3518	−0.24	Liquidambaric acid ^a^

^a^ compared with database; ^b^ compared with the literature.

**Table 3:** The binding parameters and thermodynamic parameters of the interactions between inhibitors and pancreatic lipase.

Compounds	Curcumin		Demethoxycurcumin		Bisdemethoxycurcumin	
Temperature (K)	298	310	298	310	298	310
*K_q_* (×10^12^ L·mol^−1^·s^−1^)	1.94 ± 0.10	4.28 ± 0.25	1.64 ± 0.13	3.61 ± 0.24	1.6 ± 0.07	2.97 ± 0.11
*R* ^a^	0.999	0.993	0.996	0.986	0.996	0.988
*K_a_* (×10^4^ L·mol^−1^)	1.68 ± 0.11	2.86 ± 0.14	1.20 ± 0.16	1.96 ± 0.22	1.04 ± 0.09	1.65 ± 0.13
*R* ^b^	0.998	0.987	0.996	0.999	0.996	0.997
n	1.14 ± 0.02	1.2 ± 0.02	1.12 ± 0.04	1.40 ± 0.01	1.13 ± 0.03	1.45 ± 0.02
Δ*G* (kJ·mol^−1^)	−24.11 ± 0.13	−26.44 ± 0.25	−23.28 ± 0.14	−25.48 ± 0.21	−22.91 ± 0.24	−25.02 ± 0.15
Δ*H* (kJ·mol^−1^)	−33.88 ± 0.30		−31.31 ± 0.22		−29.47 ± 0.17	
Δ*S* (J·mol^−1^·K)	−23.99 ± 0.10		−18.82 ± 0.09		−14.33 ± 0.14	

*K_q_*, the fluorescence-quenching rate constant; *R*^a^, the correlation coefficients of the *K_q_* value; *K_a_*, the Stern–Volmer binding constant; *R*^b^, the correlation coefficients of the *K_a_* value; Δ*G*, free energy; Δ*H*, enthalpy; Δ*S*, entropy change.

**Table 4:** Secondary structure analysis from circular dichroism spectra.

Samples	α-Helix (%)	β-Sheet (%)	β-Turn (%)	Random Coil (%)
Pancreatic lipase	17.7	25.4	14.8	42.1
Pancreatic lipase + curcumin	34.3	15.3	15.7	34.7
Pancreatic lipase + demethoxycurcumin	26.4	16.9	16.5	40.2
Pancreatic lipase + bisdemethoxycurcumin	21.3	21.7	15.9	41.1

## Data Availability

The data sets generated and/or analyzed during the current study are available from the appropriate authors upon reasonable request.

## Supplementary Materials

The following supporting information can be downloaded at: https://www.mdpi.com/article/10.3390/nu16152566/s1, Table S1: The common name and scientific name of 20 medicine-and-food homology plants.
