# Supplementary material for: Discovery of Curcuminoids as Pancreatic Lipase Inhibitors from Medicine-and-Food Homology Plants
Source: Nutrients. 2024 Aug 5;16(15):2566. doi: 10.3390/nu16152566 (PMC11314295; doi:10.3390/nu16152566)
Supplement: Supplementary file 1 [file nutrients-16-02566-s001.zip › nutrients-3087286-supplementary.pdf]

Table S1. The common name and scientific name of 20 medicine and food homology plants.

Samples	Scientific name	Origin	Medicinal parts
Turmeric	*Curcuma longa* Linn.	Sichuan	Root
Hawthorn Fruit	*Fructus Crataegi*	Henan	Fruit
Lotus Leaf	*Folium Nelumbinis*	Jiangxi	Leaf
Seabuckthorn Fruit	*Fructus Hippophae*	Xinjiang	Fruit
Mulberry Fruit	*Fructus Mori*	Jiangxi	Fruit
Palmleaf Raspberry Fruit	*Fructus Rubi*	Zhejiang	Fruit
Lesser Galangal Rhizome	*Alpiniae Officinarum* Rhizoma	Guangxi	Root
Solomonseal Rhizome	*Rhizoma Polygonati*	Hubei	Root
Radish Seed	*Semen Raphani*	Gansu	Seed
Mulberry Leaf	*Folium Mori*	Anhui	Leaf
Dandelion	*Taraxacum mongolicum* Hand-Mazz.	Shanxi	Entire plant
Smoked Plum	*Fructus Mume*	Jiangxi	Fruit
Citron Fruit	*Citrus medica* Linn.	Hebei	Fruit
Eucommia Bark	*Cortex Eucommiae*	Sichuan	Bark
Hyacinth Beau	*Semen Dolichoris* Album	Yunnan	Seed
Bulbus Lilii	*Lilium brownii* var. viridulum	Hunan	Bulb
Germinated Barley	*Fructus Hordei* Germinatus	Anhui	Bud
Indian Buead	*Wolfiporia cocos*	Yunnan	Sclerotium
Tall Gastrodia Tuber	*Rhizoma Gastrodiae*	Yunnan	Tuber
Cassia Seed	*Semen Cassiae*	Sichuan	Seed
